# Which Method Is More Effective for the Treatment of 1–2 cm Renal Pelvis Stones in Obese Patients: Extracorporeal Shock Wave Therapy or Flexible Ureterorenoscopy?

**DOI:** 10.7759/cureus.54194

**Published:** 2024-02-14

**Authors:** Kadir Karkin, Mubariz Aydamirov, Buğra Aksay, Eyüp Kaplan, Güçlü Gürlen

**Affiliations:** 1 Department of Urology, Health Sciences University, Adana City Training And Research Hospital Urology Clinic, Adana, TUR; 2 Department of Urology, Başkent University, Alanya Application and Research Center, Antalya, TUR; 3 Department of Urology, Health Sciences University, Adana City Training and Research Hospital Urology Clinic, Adana, TUR; 4 Department of Urology, Abdulkadir Yüksel State Hospital Urology Clinic, Gaziantep, TUR

**Keywords:** 1-2 cm calculi, pelvic stones, flexible ureterorenoscopy, extracorporeal shock wave therapy, obesity

## Abstract

Objective: This study aimed to compare the clinical outcomes and complications of obese patients who underwent extracorporeal shock wave lithotripsy (ESWL) and flexible ureterorenoscopy (FURS) for treating 1-2 cm renal pelvic stones.

Methods: This study included 89 patients with a body mass index (BMI) >30 who underwent ESWL and FURS surgeries for 10-20 mm renal pelvic stones between January 2015 and July 2023. Three months after the treatments, patients underwent full abdominal computed tomography (CT) and were examined for stone-free status and the presence of residual stones. The presence of ≥4 mm residual stones on imaging was considered a failure, and these patients were treated again. Demographic data, stone characteristics, stone-free rate (SFR) three months after the procedure, surgery/procedure time, and complications such as bleeding, urosepsis, and collecting system perforation were compared between the groups.

Results: The patients included in the study were divided into two groups: ESWL (n=46) and FURS (n=43). Demographic and clinical data were similar between the groups. Retreatment rates were higher in the ESWL group compared to the FURS group. The mean procedure time was similar between the groups (p=0.085). The three-month SFR was found to be higher in the FURS group (88.3% vs. 73.9%; p=0.043). There was no difference in complication rates between groups.

Conclusion: FURS is a more effective treatment method than ESWL in obese patients with stones 1-2 cm in size located in the renal pelvis.

## Introduction

According to the definition of the World Health Organization, a body mass index (BMI) >30 kg/m^2^ is considered obesity [1]. The incidence of obesity is increasing day by day due to changes in lifestyle and nutrition [2]. Studies have shown that obesity is an important factor in the development of urinary system stones and has been found to be a risk factor [3,4]. The treatment to be applied to these patients may be affected by comorbid diseases [5]. Extracorporeal shock wave lithotripsy (ESWL) or endourological treatments are recommended in European urology guidelines for the treatment of 1-2 cm kidney stones (excluding lower calyceal stones). ESWL holds an important place in the treatment of urinary system stone disease [6]. ESWL success is affected by parameters such as obesity, stone size, localization, and Hounsfield unit [7]. Flexible ureterorenoscopy (FURS) is also used successfully for the treatment of kidney stones due to developments in laser technology [8]. Each technique has its own advantages and disadvantages. There is no definitive consensus in the literature regarding the treatment of medium-sized (1-2 cm) kidney stones. This study aimed to compare the clinical outcomes and complications of obese patients who underwent ESWL and FURS due to 1-2 cm renal pelvic stones.

## Materials and methods

Our study was approved by the Ministry of Health and the local ethics committee (Approval Number: 11.05.2023/2968). Our study included 89 patients with a BMI >30 who underwent ESWL and FURS surgeries for 10-20 mm renal pelvic stones between January 2015 and July 2023. Cases with kidney anomalies, bleeding disorders, ureteral obstruction, positive urine culture, pregnant patients, kidney failure (serum creatinine ≥1.5 mg/dL), and those with a history of previous unsuccessful stone treatment were excluded from the study. Urine analysis, urine culture, hemogram, and kidney and liver function tests were performed on all patients before surgery. The procedure was planned after appropriate antibiotic treatment was given to patients with growth in urine culture. Whole abdominal non-contrast computed tomography (CT) was performed as an imaging method to detect the size, number, and location of the stones. Prophylactic intravenous broad-spectrum antibiotic therapy was administered before FURS surgery. The procedures for patients in the ESWL group were performed by an experienced technician using an electrohydraulic lithotripter (EMD E-1000, Ankara, Turkey) under the supervision of a urologist. The procedures were performed as outpatient treatment under intravenous sedation anesthesia, in the supine position, and under fluoroscopy guidance. Sessions were applied at 80 shocks per minute, with a maximum of 3000 shocks. ESWL was applied again to those with ≥4 mm residual stones on control imaging (direct urinary system radiography or CT) 10 days after the procedure.

In the FURS group, the procedure was performed by two surgeons in the lithotomy position under general anesthesia. First, entry was through the urethra with a rigid ureteroscope. A zebra guide wire (0.038 mm) was sent to the renal pelvis through the orifice on the side with the stone. A ureteral access sheath (UAS) (Olympus uropass ureteral access sheath 12/14F x 38 cm) was placed in the proximal ureter over the guide wire. In cases with narrow ureters and in cases where UAS could not be inserted, a double-J stent was emplaced, and the procedure was postponed for one month. The guide wire was removed, and a flexible ureteroscope (Olympus URF-P5 Flexible ureteroscope, Tokyo, Japan) was sent to the pelvis through the UAS. The stones were broken using a 200 μm holmium laser. After the fragmentation was completed, control fluoroscopy was used to determine whether any residual stones remained. A double-J stent was routinely inserted in all patients after the procedure, and the procedure was repeated in the presence of ≥4 mm residual stones on control imaging one month after the surgery, and the double-J stent was removed in those with no residue. Three months after the treatments, patients underwent full abdominal CT and were examined for stone-free status and the presence of residual stones. The presence of ≥4 mm residual stones on imaging was considered a failure, and these patients were treated again. Demographic data, stone characteristics, stone-free rate (SFR) three months after the procedure, surgery/procedure time, and complications such as bleeding, urosepsis, and collecting system perforation were compared between the groups. For ESWL, the period from the first shock application to the last shock application was determined as the procedure time. For FURS, the period from entry into the urethra with the ureteroscope until the placement of the urethral catheter was considered the surgery time.

Statistical analysis

Analysis of data was performed using IBM SPSS Statistics for Windows, Version 16 (Released 2007; IBM Corp., Armonk, New York). Student's t-test and chi-square test were used to analyze data between groups. Categorical variables are expressed as frequency (%) and continuous variables are expressed as mean±standard deviation (SD). A value of p<0.05 was considered statistically significant.

## Results

**Figure 1:**
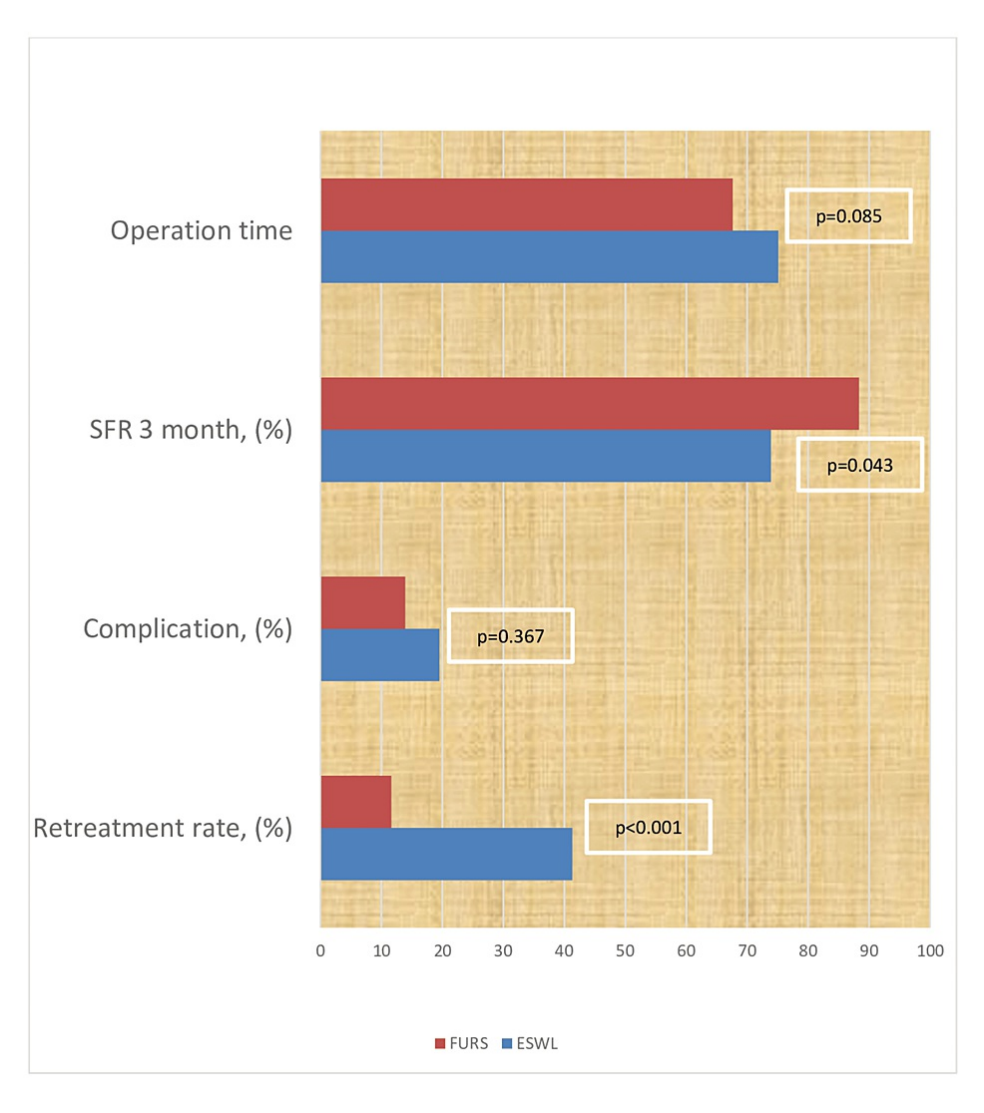
Comparison of treatment, post-treatment success, and complication rates

The patients included in the study were divided into two groups: ESWL (n = 46) and FURS (n = 43). Demographic and clinical data were similar between the groups (Table 1). Treatment and post-treatment data are summarized in Figure 1. Retreatment rates were higher in the ESWL group compared to the FURS group (41.3% versus 11.6%, respectively). Since a ureteral access sheath (UAS) could not be inserted in five patients in the FURS group, a double-J stent was emplaced, and the procedure was postponed. The mean procedure time was similar between the groups (p=0.085). The three-month SFR was found to be higher in the FURS group (88.3% vs. 73.9%; p=0.043). There was no difference in complication rates between groups. In the FURS group, one patient developed urosepsis and recovered after treatment, and five patients had postoperative fever and were treated with appropriate antibiotics and antipyretics. In the ESWL group, three (6.5%) patients developed Steinstrasse, and four patients experienced fever. One of the cases with Steinstrasse underwent ureteroscopic lithotripsy surgery, and the others passed their stones during follow-up. Gross hematuria was observed in two patients. The patients were hospitalized, and hematuria was controlled with medical treatment.

**Table 1:** Comparison of patients' demographic and clinical data HTN, hypertension; DM, diabetes mellitus; BMI, body mass index; ESWL, extracorporeal shock wave lithotripsy; FURS, flexible ureterorenoscopy.

Demographic and clinical data	ESWL (n=46)	FURS (n=43)	p
Age, mean±SD	39.2±14.4	35.6±12.1	0.214
Gender, n (%)			0.871
Male	28 (60.1)	27 (62.8)	
Female	18 (39.9)	16 (37.2)	
BMI, mean±SD	37.1±4.3	38.4±3.8	0.116
DM, n	7	6	0.457
HTN, n	9	7	0.342
Side of stone, n (%)			0.863
Right	26 (56.5)	24 (55.8)	
Left	20 (43.5)	19 (44.2)	
Stone size, mean±SD	17.3±2.2	18.1±1.5	0.724

## Discussion

In this study, FURS and ESWL methods were investigated to determine which was more successful for the treatment of 1-2 cm renal pelvic stones in obese patients. Although surgery times and complication rates were similar for both methods, retreatment rates were found to be higher in ESWL patients compared to FURS, and the SFR was found to be lower after three months. Up to 5-15% of the world's population is affected by urolithiasis [9]. The increase in obesity rates has led to an increase in the prevalence of stone disease [10]. Studies show that the prevalence of stone disease is twice as high in obese patients compared to non-obese patients, and recurrence rates are also high [11]. The success of ESWL depends on stone (localization, size, Hounsfield unit, skin-stone distance) and patient-related factors (BMI, skeletal anomaly) are also effective. ESWL is more likely to fail, especially in those with a skin-stone distance (SSD) of 10 cm or more [12]. Similar to the results of this study, Pareek et al. stated that endoscopic surgery may be more appropriate for those with a BMI >30 kg/m^2^ [13].

Studies have shown that obesity does not affect the results of FURS surgery, unlike ESWL [14,15]. In a meta-analysis, an 87.5% SFR was found in obese patients undergoing FURS [16]. In the meta-analysis results by Mi et al., where they examined 2348 patients, for 1-2 cm kidney stones, FURS was found to have higher SFR and lower additional intervention and retreatment rates than ESWL [17]. With similar complication rates, this study found that the FURS group was advantageous compared to ESWL in terms of SFR and retreatment rates. However, the literature indicates that there is no difference between the two methods in terms of SFR for stones <1 cm [18]. In their study with 46 obese patients, Javanmard et al. found that the three-month SFR after ESWL was 68% [19]. In this study, the three-month SFR rate in the ESWL group was 73.9%. In our study, the three-month SFR in the FURS group was 88.3%, and this was compatible with the literature.

Although complications are a significant concern, they are generally managed with medical treatment after both methods. Recent technological developments in FURS have increased the safety and effectiveness of the procedure [20]. The use of a ureteral access sheath is extremely important as it provides easy access to the kidney with the flexible ureteroscope, reduces intrarenal pressure, contributes to the active removal of broken stone fragments, and increases image quality due to continuous irrigation. Despite these advantages, injuries to the ureter and stenosis in the postoperative period are observed due to the use of a ureteral access sheath [21]. In some clinics, double-J stents are routinely inserted before FURS surgery. In this study, double-J stents were not placed routinely, and no stenosis development was observed during the follow-up of the patients.

Another important issue is the complications that may occur due to the procedures. One of the most significant complications in ESWL is Steinstrasse, observed at a rate of 2% to 10% [22]. As the size of the stone increases, so does the incidence of Steinstrasse. While some patients can pass these stones with medical treatment, additional surgical interventions may also be required. Although the SFR was low and retreatment rates were high in the ESWL group, it is more frequently preferred by patients and doctors as it is a non-invasive treatment method. Since FURS surgery can be performed successfully in a single session, it is advantageous for patients who do not want to experience colic-like pain during repeated ESWL sessions. The main disadvantages of FURS are high stone volume, the presence of multiple stones, long operating time depending on the surgeon's experience, and high operating costs. For many reasons like these, FURS is not performed in many centers. According to the results of this study, FURS is safer and more successful in obese patients with ≤2 cm kidney stones.

Our study has some limitations. The main limitations are the small number of patients, the study being conducted retrospectively, and the lack of cost analysis, subgroup analysis of obese patients, skin-stone distance measurements, stone analysis results, information about analgesic use between groups, and pain score examination.

## Conclusions

According to the results of our study, the outcomes of FURS surgery appear to be better in obese patients with stones 1-2 cm in size located in the renal pelvis. An individual approach to patients is important, taking into account the patient's expectations, comorbid diseases, treatment duration, and costs.
